# Genetics of Dyslipidemia

**DOI:** 10.5935/abc.20160074

**Published:** 2016-05

**Authors:** Diego García-Giustiniani, Ricardo Stein

**Affiliations:** 1 Health in Code, La Coruña, Spain; 2 Grupo de Investigación Cardiovascular de La Universidad de La Coruña, La Coruña, Spain; 3 Grupo de Pesquisa em Cardiologia do Exercício do Hospital de Clínicas de Porto Alegre, Porto Alegre, RS - Brazil; 4 Serviço de Cardiologia do Hospital de Clínicas de Porto Alegre - Universidade Federal do Rio Grande do Sul, Porto Alegre, RS - Brazil; 5 Vitta Centro de Bem Estar Físico, Porto Alegre, RS - Brazil

**Keywords:** Dyslipidemias / genetics, Hyperlipoproteinemia Type II, Dyslipidemias / diagnosis

Lipoproteins constitute a set of proteins and lipids, organized to facilitate the transport of lipids through blood plasma. Elevated or decreased levels of these lipoproteins may be related to genetic alterations in 40% to 60% of cases.^1^ This fact explains why it is common to find lipid abnormalities in several members of the same family. The elevated levels of these composite organic biomolecules are responsible for approximately 50% of the attributable risk for the development of atherosclerotic cardiovascular diseases,^2,3^ a process in which other phenotypes with a hereditary component also participate, such as diabetes, obesity and metabolic syndrome.

Population studies of genetic association identified more than one hundred genes that could have a direct impact on lipid levels.^3^ These genes affect plasma levels of total cholesterol, low-density lipoprotein-cholesterol (LDL-c) and high-density lipoprotein cholesterol (HDL-c), as well as of triglycerides, being capable of establishing complex phenotypes. However, among these genes identified in the association studies, there is a previously known and well-characterized group of genes responsible for the development of monogenic dyslipidemias. In such cases, the variant in a single gene clearly explains the phenotype.

It is noteworthy that even when isolated, these mutations determine diseases considered rare and the impact produced by variants of these genes tend to be high, resulting in extreme values of lipid levels. Considering that cardiovascular atherosclerotic disease is a process that starts in childhood and progresses throughout life,^4^ the early identification of risk factors is very important. In this sense, the diagnosis of phenotypes determined by monogenic diseases of lipid metabolism deserves special attention, considering that, when they produce extreme phenotypes, they can be associated with the development of early atherosclerosis of rapid progression.

In fact, genetic studies using Next-Generation Sequencing (NGS), carried out in patients with early acute myocardial infarction (AMI) (that occurring before 50 years of age in men and 60 in women) have determined that the presence of rare genetic variants in the LDLR gene resulted in a 4-fold higher risk of this event in their carriers. On the other hand, the rare variants in the APOA5 gene, associated with high triglyceride levels, resulted in a 3-fold higher risk. Moreover, approximately 2% of the cases were carriers of a clearly pathogenic mutation in LDLR, suggesting that these patients had a diagnosis of familial hypercholesterolemia.^2^ In a broader sense, it is estimated that approximately 5% of AMIs in patients aged < 60 years may be due to familial hypercholesterolemia, a figure that can increase up to 20% when acute coronary events affect individuals younger than 45 years.^5^

It is noteworthy that even in the case of monogenic diseases, mutations identified in many of the genes frequently responsible for these diseases show significant variability in phenotypic expression and the associated risk, either in carriers from the same family or among carriers of the same mutation that belong to different populations.^6-9^ Occasionally, this fact may hinder or delay diagnosis in patients to whom only clinical criteria are applied, since in those circumstances, lipid levels may overlap those observed in the general population.

The great variability in clinical expression in patients with a certain variant is due to the effect of other factors, which would act as modifiers for the expression of the accountable mutation, a fact that has been demonstrated in several occasions. Incidentally, this is the case of variants in genes such as ApoB, PCSK9, LRP1 and Lp(a), as well as in carriers with mutations in LDLR diagnosed with familial hypercholesterolemia, just to name a few examples.^6-11^ The participation of genetic variants as phenotypic expression modifiers is explained by the fact that the genes involved can be independently associated, being capable of promoting an increase or decrease in the lipoprotein levels that they affect. In other words, the interactions of their effects attenuate or increase lipid levels, as well as the associated risk in carriers. Similarly, certain variants may influence not only the phenotypic expression, but also the response to drug treatment, determining that the intervention be or not effective in its goal of reducing the associated risk.^6,7^

## Genes Associated with Alterations in LDL-C Levels

### Familial Hypercholesterolemia

This familial disease is one of the most prevalent monogenic disorders, being identified in approximately 1 in 500 individuals in the general population. Interestingly, in some European subpopulations, it has been estimated that the frequency is even higher, around 1 in 250 individuals.^8^ In the past, the characterization of familial hypercholesterolemia was carried out using only clinical and laboratory criteria. However, knowledge of this strategy limitations, regarding its low sensitivity for the diagnosis,^9,10^ motivated changes in two of the three main diagnostic criteria of this disease entity.

According to the criteria established by the Dutch Lipid Clinic Criteria (Table 1) and the Simon Broome Diagnostic Criteria, the identification of pathogenic mutations, when added to additional clinical criteria, has been used to consider the diagnosis of familial hypercholesterolemia as a definitive one.

**Table 1:** Dutch Lipid Clinic Criteria for the diagnosis of familial hypercholesterolemia (score)

Criteria			Score
**Family history**			
First-degree relative with early coronary disease (men < 55 years and women < 60 years)			1
First-degree relative with LDL-c levels > 210 mg/dL			
First-degree relative with tendon xanthomas and/or corneal arc < 45 years			2
Family member < 18 years with LDL-c ≥ 150 mg/dL			
**Personal history**			
Patients with early coronary disease (men < 55 years and women < 60 years)			2
Patient with early cerebrovascular or peripheral artery disease (men < 55 years and women < 60 years)			1
**Physical examination**			
Tendon xanthomas			6
Corneal arc in individuals < 45 years			4
**Laboratory analysis**			
LDL-c ≥ 330 mg/dL			8
LDL-c 250-329 mg/dL			5
LDL-c 190-249 mg/dL			3
LDL-c 155-189 mg/dL			1
**Genetic analysis**			
Functional mutation in one of these genes: LDLR, APOB or PCSK9			8
**Diagnosis of familial hypercholesterolemia**			
Definite diagnosis: ≥ 8 points	Probable diagnosis: 6-7 points	Possible diagnosis: 3-5 points	

LDL-c: low-density lipoprotein-cholesterol.

Incidentally, the identification of pathogenic mutations in probands constitutes the first step to start the genetic screening in this individual's family. This approach is of utmost importance in pediatric and adolescent family members, in which the clinical criteria may not be well defined, although the disease may have already started to exert its effects at the vascular level. Regarding the diagnosis and management of this disease, such perspective has been supported by guidelines established by important medical societies, such as the Canadian,^11^ the British (National Institute for Health and Clinical Excellence -NICE),^12^ the Australian / New Zealander^13^ and the Spanish society,^14^ among others.

The genes for which there is demonstrated evidence of association with familial hypercholesterolemia are LDLR, ApoB, PCSK9 and LDLRAP1. The main lipoprotein alteration associated with the phenotype is the increase in the LDL-lipoprotein levels. However, some variations in the ApoB and PCSK9 genes may determine the decrease of this lipoprotein. It is noteworthy that the phenotype determined by the three genes, LDLR, ApoB and PCSK9, shows a pattern of autosomal dominant transmission, while the phenotype determined by mutations in the gene LDLRAP1 has an exclusive autosomal recessive transmission.

### Phytosterolemia

This is one of the main differential diagnoses in patients with suspected familial hypercholesterolemia. This disease is caused by the abnormal absorption of cholesterol and accumulation of sterols, especially those of plant origin (hence its name). The clinical features of this disease include the presence of xanthomas and premature coronary atherosclerosis, as well as hemolytic anemia and/or liver disease. The genes associated with this disease are ABCG5 and ABCG8. Regarding other genes that alter the metabolism of this lipoprotein, it includes a group associated with a decrease in its levels (familial hypobetalipoproteinemia), with a possible protective effect for cardiovascular disease in carriers.^15^ The genes associated with these phenotypes are ANGPTL3, MTTP and MYLIP.

### Genes Associated with Triglyceride Metabolism Alterations

The role of hypertriglyceridemia caused by genetic factors as a risk factor for atherosclerotic cardiovascular disease has been well established. In a recent study, which included a large cohort of patients with early AMI (before age 50), in which sequencing was carried out through NGS, carriers of APOA5 gene variants had an increased risk up to 3.3-fold higher when compared to non-carriers.^16^ A similar association had been previously established in another large cohort (the Copenhagen Cohort), in which the LPL gene was also investigated. In this observational study, it was determined that for every increase of 1 mmol/L (39 mg/dL) in triglyceride-rich cholesterol (remaining), which is mediated by genetic cause, an increase in risk for coronary artery disease (CAD) was 2.8 higher than in controls.^17^ The main genes associated with increased levels of triglycerides (as main phenotype) are APOA5, APOC2, APOE, GPD1, GPIHBP1, HNF1A, LMF1, LPL and SLC25A40. It is noteworthy that a group of rare variants in the APOC3 gene was associated with a reduction in triglyceride levels, promoting reduction of up to 40% in the risk of CAD.^18^

### Genes related with HDL-c levels

Epidemiological studies of the 1990s established an inverse association between HDL-c levels and CAD risk.^19^ At present, what is known is that this association seems to be more related to the quality of the HDL-c particles, due to structural abnormalities secondary to mutations, than the absolute amount of this lipoprotein^20,21^ - a fact that may explain the recent questions about the meaning of HDL-c levels on the development of CAD.^22^

The typical example of the structure/function association is that represented by a mutation affecting apolipoprotein A1 (ApoA1 ratio), a major constituent of HDL lipoprotein, which is associated with an effect opposite to that expected. For instance, the mutation (Arg173Cys) determines the production of ApoA1 dimers, determining that HDL-c levels be extremely low. However, these particles are very efficient in reverse cholesterol transport, which makes the carriers have a very low incidence of ischemic heart disease, although an extremely low quantitatively measured HDL-c.

Nevertheless, low levels of HDL-c of monogenic etiology determine a group of diseases that have a high prevalence of early atherosclerotic cardiovascular disease. The genes associated with low HDL-c of monogenic etiology are APOA1, ABCA1, LCAT, SAR1B and ABCG1.

APOA1 is the gene responsible for the phenotype of familial hypoalphalipoproteinemia, an entity with autosomal dominant etiology. The ABCA1 gene, in turn, is associated with Tangier disease, which is autosomal recessive. Similarly, the LCAT genes, which are associated with fish-eye disease, and the SAR1B gene, which is related to chylomicron-retention disease, also occur with decreased levels of HDL-c.

On the other hand, there is a group of genes associated with increased levels of HDL-c. Nevertheless, there is no evidence of a protective effect against atherosclerosis. One explanation for this finding might be related to the fact that these genetic variants alter the distribution of subclasses of HDL-c particles, increasing the less protective ones.^23^ This information may be relevant to determine whether the increase of this lipoprotein may be considered when calculating the cardiovascular risk associated with the lipoprotein ratios. The genes associated with increased HDL-C of genetic etiology are CETP, LIPC, PLTP and SCARB1.

### Secondary Causes of Dyslipidemia

#### Lipodystrophies

Lipodystrophies constitute a heterogeneous group of rare diseases of which common characteristic is the selective loss of adipose tissue. However, they predispose to the development of metabolic complications similar to those seen in obese individuals. Among these, are alterations in the lipid metabolism (increased triglycerides and reduced HDL-c) as well as insulin resistance and diabetes mellitus, entities associated with increased risk of premature atherosclerosis.^24,25^ The main genes associated with these diseases are LPL, AGPAT2, BSCL2, CAV1, CIDEC, LMNA, PLIN1, PPARG, PTRF and ZMPSTE24.

### Other Genes of Interest

#### Statin-induced myopathy

Muscle pain secondary to treatment with statins is the adverse effect most often associated with the use of these drugs. It is estimated that it affects 1 to 10% of treated patients,^26^ being an underdiagnosed complication.^27^ The severity and risk for the development of major complications, such as rhabdomyolysis, are related with the combined use of statins, dose and factors such as age and gender, as well as the concomitant use of other drugs affecting the bioavailability of these compounds.

The genetic determinants of associated risk consist of genes encoding enzymes related to the metabolism of these drugs, as well as carriers and genes associated with mitochondrial dysfunction. All these factors add up to those effects caused by statins themselves, as a cause of muscle damage.^28^ The genes with the highest level of association evidence with this complication are AMPD1, COQ2, CPT2, CYP2D6, PPARA, PYGM, SLC22A8 and SLCO1B1.

## Conclusion

Primary dyslipidemias or those without apparent cause can be classified genotypically or phenotypically through biochemical analysis. In genotypic classification, dyslipidemias are divided into monogenic (caused by mutations in a single gene) and polygenic (caused by associations of multiple mutations, which would not be of great impact when alone).^29^

The knowledge of the molecular basis of dyslipidemias allows their correct diagnosis. In this scenario, the institution of an optimal drug therapy may be better founded. Moreover, genetic diagnosis in an index case can trigger a family investigation process, which allows early detection, therapeutic guidance and consequent reduction of cardiovascular risk in such individuals.
